# Remarks on an East-Indian Medicine, Termed Nannari, or Country Sarsaparilla

**Published:** 1831-03

**Authors:** John Ashburner


NANNARI.

### *Remarks on an East-Indian Medicine, termed Nannari, or Country Sarsaparilla.* By John Ashburner, m.d.

*To the Editor of the London Medical and Physical Journal.*

Sir: An excellent and enterprising friend of mine, in the East Indies, some time ago sent to England a quantity of a medicinal agent, well known on the Malabar coast, under the name of Nannari, or country sarsaparilla. The substance has here excited but very trifling notice among medical men, although, from several striking properties which it possesses, it is certainly not unworthy of attention. From the few opportunities I have had of trying it, I should, I think not too hastily, conclude that it may become a very valuable substitute for sarsaparilla. It possesses, undoubtedly, more decided power than that substance, although it may be a question whether its force can be directed in the same trains. In India, I heard it spoken of as a purifier of the blood, and as possessing power over the functions of the stomach; and learned that it was in repute among the native doctors, as a remedy in many obstinate diseases of the skin: but my particular attention never having been then called to the examination of the material, I cannot remember its external characters, or recall my impression as to whether they corresponded with the description of the Periploca Indica, given in an admirable work, first published at Madras in the year 1813, by Dr. Whitelaw Ainslie, and lately much enlarged by him in this country, under the title of Materia Indica. No one who has not had occasion to dive into the medical recesses of Indian bazaars, can have an adequate idea of the value of this work: it is a monument of learning, and of vast industry, which must attract for the name of its author the admiration of all men who can appreciate useful knowledge, who delight in curious information, and who have respect for occupations that are at the same time virtuous and laborious. The book ought to be in the hands of every person who thinks of going to India.

The account which Dr. Ainslie gives of, what he calls, Country Sarsaparilla, or Nunnarivayr, or Periploca Indica, differs from that which I should give of the substance that has been sent to England from the Malabar coast; and there is reason to believe that this is Dr. Ainslie's Cari Vilandi, or Smilanga of the Greeks. I have shewn the substance to several medical friends, who are about to make trial of its powers. Mr. Garden, of Oxford street, and some other druggists, have supplied themselves with the article; and I trust that the worthy and spirited merchant, who first sent the drug to England, will have more than the satisfaction of having benefited his country by the introduction of a material which may prove a beneficial substitute for a very expensive medicine. Too often, to my knowledge, have well-intentioned adventurers had occasion to sing Virgil's stanza: "Sic vos non vobis."

Professor THOMSON, of the London university, has, among others, seen the substance I now bring to your notice, and which, from Ainslie, I had ventured to name Smilax Aspera, and he thinks that it belongs to the natural order Smilaceæ. He describes it thus: "Roots long, very tortuous, cylindrical; in thickness varying from the size of a crowquill to one third of an inch in diameter, sparsely furnished with lateral fibres. The root consists, as in the Smilaces, of a central woody part, or medulla, which is covered with a thick spongy bark, contracted at irregular intervals, so as almost to appear *moniliform*, the contracted parts being cracked down to the medulla. The epidermis is of an ashy, reddish hue, corrugated in longitudinal furrows; the cortical part is whitish yellow; the fracture is short and starchy." To this account I should add, that the odour is fragrant, aromatic, between that of brandy and peach-blossom. The taste, when chewed, is slightly sweetish, spicy, and rather bitter; the woody fibre is tough. Mr. Garden has been making some experiments with this substance, and has, at my request, been so obliging as to furnish me with the following notes of them:

A. Four ounces of the bruised root of the Smilax aspera were mixed with sixteen ounces of distilled water, then introduced into a glass retort, to which a receiver was adapted, and submitted to distillation, upon a moderately heated sand bath.

The liquid which distilled over into the receiver had a slightly aromatic and cooling taste, and was strongly impregnated with the peach-blossom odour of the root: it reddened very distinctly the blue colour of litmus paper, but did not exhibit any of the well-known characters of hydrocyanic acid. The contents of the retort were washed out with an additional quantity of distilled water, and exposed for some time to a boiling heat, in a porcelain vessel. The liquid was then strained off, and a fresh quantity of water added to the root, with which it was again boiled, and strained off as before. In this manner the root was treated with successive portions of boiling water, until this fluid ceased to acquire any further colour or taste from the root.

The whole of these decoctions were now mingled together, and strained through a linen cloth, and gradually evaporated to the consistence of honey. A quantity of extract was thus obtained, equivalent to one fourth of the weight of the root employed, namely, one ounce. This extract possessed all the sensible properties of the root: it had a very pleasant and fragrant smell, a distinctly bitter and agreeable aromatic taste, and communicated at the same time a slight sensation of sweetness to the palate.

B. An indefinite quantity of the bruised root was repeatedly digested in several successive portions of alcohol, of the specific gravity .830, until that fluid no longer extracted any thing from the root. The alcoholic infusions were all mingled together, and filtered through paper. The liquid had a pale amber yellow colour, and was sensibly impregnated with the peculiar fragrant odour of the root.

The whole of the liquid was poured into a glass retort, connected with a receiver, and exposed to distillation in a water bath, when about two thirds of the alcohol had distilled over. The liquid remaining in the retort was poured into a porcelain capsule, and suffered to cool. At the end of twelve hours, a considerable quantity of a granular and semi-crystalline-looking substance, of a white colour, was found deposited upon the bottom and sides of the capsule. The supernatant liquid was poured off, and exposed to spontaneous evaporation for some time in a warm atmosphere, by which means a further quantity of solid matter, similar in appearance to the last, was separated.

The fluid which now remained was of a brown colour, and of the consistence of syrup. It was put aside for further examination.

The solid substance which had been deposited by evaporating the alcohol, was collected together, and pressed betwixt several folds of blotting paper, so as to absorb all adhering moisture: when dry, it exhibited the following characters.

Tasteless and inodorous; softened by a very gentle heat, and adhered to the fingers in the manner of resin; fusible at a temperature of 260° Fahr.; and at a still higher heat it kindled, and burned with a dull flame, emitting much smoke, but leaving no solid residue after combustion.

Boiling water softened, but did not dissolve it. Alcohol at common temperature dissolved it sparingly, but, when heated, dissolved it with considerable facility, and deposited it again upon cooling. Sulphuric æther, at common temperatures, dissolved it very readily. This substance was also perfectly dissolved by the fixed oils, and by the oil of turpentine.

Neither the alcoholic nor æthereal solutions produced any change in the blue colour of litmus paper, or the yellow colour of turmeric paper.

From the preceding results, the substance in question appears evidently to be of a resinous nature; but as its fusing point is not very high, and as it is but little soluble in cold alcohol, it must be considered as differing in some degree from common resin.

Its prominent characters may be regarded as intermediary betwixt those of resin and wax.

The brown-coloured liquid which remained after the evaporation of the alcohol, and which had been set aside with a view to see whether any further separation of solid matter would take place, was gradually evaporated to the consistence of honey, and put aside for a few days in a covered vessel. At the end of this period, no change in its appearance had occurred.

It possessed the odour of the root in an eminent degree; communicated to the palate a strong bitter taste, mingled with a not unpleasant aromatic sweetness.

In warm water it was readily soluble, to the exception of a few flakes of resinous matter, which separated upon cooling. This aqueous solution reddened very strongly the blue colour of litmus paper.

From the foregoing statement, it becomes exceedingly probable that the active principle of the Smilax asperia will be found to reside in the brown-coloured uncrystallizable liquid which remains after the resinous substance has been separated from the alcoholic solution.

That it appears to possess acid, and not alkaline properties, is shewn from the effect produced on the blue colour of litmus paper.

The quantity of this substance upon which I have yet operated, has not been sufficient to enable me to determine whether the acid properties which it exhibits be due to the presence of an acid *sui generis*, or to some one of the vegetable acids already known, associated with a bitter principle. At a future day I hope to pursue the inquiry.

Dear sir, yours very truly, A. GARDEN.

I shall conclude this notice by some observations I have made on the Nannari, as a medicinal agent. In the few cases in which I have given it, the form of exhibition has been that of decoction. I have directed two ounces of Nannari to be boiled in a pint and a half of water, with a drachm of extract of liquorice and half a drachm of subcarbonate of soda; the decoction has been simmered to a pint, and strained. This pint has been taken at intervals in the course of the day.

I have had several opportunities of administering this decoction, and the most striking effect has appeared to me to be its action upon the kidneys; attended, however, with a previous gnawing sensation of the stomach. I select two of them:

A gentleman, forty-two years of age, of dark complexion, brown hair, hazle eyes, short stature, had been many years engaged in mercantile occupations. He has relinquished his former pursuits for several years, and, having a turn for convivial enjoyments, has indulged in the pleasures of the table. His account was, that he had never been in the habit of drinking to excess, but he was sensible that he had long daily taken more than was good for his clearness of head, or for his general health. I found him, three months ago, with a troublesome cough, copious expectoration, great emaciation, and want of strength; an anxiety of countenance, and hurried temper. The appetite was bad; digestion was performed with much irregularity; the bowels were moved with purgatives, but he seldom evacuated without medicine. The motions were ash-coloured and offensive; the urine was scanty; the mouth was clammy; the tongue was furred, and coated with a dark yellow crust: the fauces were red, and he complained of constant roughness at the back of his throat. The most distressing circumstance in the case was, however, the very copious perspiration he experienced in the night, which caused the bed to be drenched with moisture. He had a constant dull pain in the right hypochondrium, much increased upon pressure under and along the margin of the ribs. An occasional pain in the head troubled him, which was attended by extremely cold feet. This gentleman had taken various remedies that were indicated by his symptoms, without effect: blue pill and purgatives, nauseating doses of antimony, sulphuric acid, with bitters and neutral salts. I directed a seton near the hypochondriac pain; bluepill and calomel combined, to be taken every other night; and a pint of the decoction of Nannari daily. The diet consisted of broths, milk in various forms, toast and tea. The effect of the treatment was very beneficial: the cough and expectoration were daily less troublesome. A gnawing hungry sensation at the pit of the stomach came on. A diuresis supervened, obliging him to have recourse very repeatedly in the night to the urinal. It was at the end of a fortnight that his night-sweats had ceased, and, after taking the decoction of Nannari daily for nearly six weeks, he found himself with improved appetite, and a condition of evacuations that was quite healthy. The mercurial doses had not been pursued regularly, but had been used as evacuants.

Another case, illustrative of the action of Nannari on the kidneys, occurred under my observation about the same time. A lady, sixty-two years of age, had been for six years subject to swelled legs; the ankles were œdematous. She was a stout tall woman, of sallow complexion, with dark eyes. Twelve years ago she had psoriasis on her hands, elbows, and knees, with some pityriasis about her neck, which I had in vain endeavoured to cure: they vanished, for time had done more than the doctor. For the last two years, a difficulty of breathing has assailed her in every effort she has made to mount a stair; and the impeded circulation is apparent on such occasions, from the livid lips and difficulty of respiration. She has had a sense of weight in her loins, and a weariness whenever she extended her walks beyond a few hundred yards from her door. Dinner pills, blue pills, senna draughts, infusions of rhubarb and gentian, with alkalies, changed occasionally for neutral salts, have been duly swallowed, in pursuance of advice from the learned. These have tended to regulate the alvine evacuations; but an unconquerable sleepiness after dinner, a sense of oppression and weight in the epigastrium after every meal, were very troublesome to the patient. She made an unusually small quantity of water, and as the legs enlarged, she became alarmed. Various diuretics were prescribed, without much benefit. I mentioned the Nannari, and recommended a trial of it. A pint of the decoction daily, in three days, produced a desire to eat luncheon, which had not been experienced for years. A few days afterwards, my patient told me she was voiding an unusual quantity of urine, and that the legs felt much lighter. This beneficial effect made her very happy for a fortnight, during which time the ankles regained their natural condition. I am sorry I cannot say that the remedy continues its beneficial influence : the similarity of its effect, however, in several other cases to that which resulted in these, is sufficient to make me feel warranted in drawing the attention of the medical public to a trial of its efficacy.

I have exhibited the remedy in no other form besides the decoction. The infusion in lime water makes an elegant preparation, and the syrup and extract might be very convenient modes of administering it in combination with alkalies, neutral salts, or other adjuncts.

I am, sir, your obedient servant,

JOHN ASHBURNER.

5, *Wimpole street; January* 1831.

